# Low retention rate of voluntary blood donors: contribution of an original method based on a composite classification (results of a monocentric study in the Democratic Republic of Congo)

**DOI:** 10.11604/pamj.2020.36.296.24714

**Published:** 2020-08-18

**Authors:** Susanne Mbaka Ngunza, Cyprien Munyashongore, Gisèle Nshokano Nshobole, Dominique Latine, Isabelle Aujoulat

**Affiliations:** 1 Cliniques Universitaires de Bukavu, Université Officielle de Bukavu, Bukavu, République Démocratique Congo,; 2 Institut de Recherche Santé et Société, UCLouvain, Bruxelles, Belgique,; 3 Ecole de Santé Publique, Université Nationale du Rwanda, Kigali, Rwanda,; 4 Centre Provincial de Transfusion Sanguine, Bukavu, République Démocratique du Congo,; 5 Institut de Recherche Expérimentale et Clinique, UCLouvain, Bruxelles, Belgique

**Keywords:** Low retention rate, voluntary donors, blood

## Abstract

**Introduction:**

in order to improve the safety of blood transfusion, the retention of voluntary donors remains a major concern in the Democratic Republic of Congo. Nevertheless, retention is still difficult to assess because of the lack of local studies. The present study establishes the donors' profile and regularity, as well as regularity-associated factors, at the Provincial Blood Transfusion Centre in Bukavu.

**Methods:**

this descriptive and analytical cross-sectional study included the records of 387 out of 773 blood donors during the period from 2015 to 2017. Donor retention and its associated factors were measured. The composite approach used here considered the number of blood donations, their frequency, the previous regularity of donors and the inter-donation interval.

**Results:**

we bring to light an important loss of regular voluntary donors in the centre. Only 23.8% of them were still regular donors in 2017. The majority of donors registered in the centre are young males and have no income. On the contrary, factors associated with the profile of a regular donor in 2017 were: age at least 46 years old, being a woman and working in the formal sector. The composite classification highlighted that an important proportion of former regular donors, namely 72.8% (N=161/221), had not given blood in 2017.

**Conclusion:**

the use of a composite classification to assess the regularity of voluntary blood donors provides more accurate information that will enable the improvement of donors' awareness and retention as well as the possible reinstatement of former donors.

## Introduction

Transfusion-associated care occupies a special place in modern health care [1] but the supply of voluntary donations, considered as the only safe source of blood, remains a major concern in developing countries, especially in sub-Saharan Africa and in the Democratic Republic of Congo (DR Congo) [2]. Ensuring a regular supply requires the recruitment and retention of voluntary blood donors [2-4], as replacement donations (family and paid) fail to ensure a sustainable and regular blood supply [2]. Moreover, these replacement donations increase the risk of transfusion-transmitted infections (TTIs) for the recipient, especially in our context of scarce resources [2,5,6]. The rate of voluntary donations is low in Africa, as well as in the African populations living in developed countries [2,7]. Only 0.5% of the population donates blood on a voluntary basis in sub-Saharan Africa, i.e. half of the minimum preconised by the World Health Organisation (WHO) to meet the needs [2]. The rate falls to 0.34% in DR Congo, where only 70% of transfusional requirements are met, essentially through replacement blood donations (66%) [2-4,8]. Maintaining an adequate flow of blood donations in order to adresse the shortages requires the retention of recruited voluntary donors and an increase of donations by existing donors [9]. To address such a two-fold challenge, blood donation establishments and centres must know their donors, their particularities as well as the frequency and motivation to donate blood [10,11]. Such knowledge is an essential prerequisite for a pertinent communication during awareness campaigns aiming at increasing the retention of voluntary donors. However, the assessment of donors’ frequency and regularity-associated factors remains a problem.

Two existing classifications allow assessing the regularity of voluntary blood donors [12], but none is entirely satisfactory to date. The first approach defines regularity according to the number of donations, regardless of the last donation date, which is confusing as the threshold to define a donor as regular is quite variable [12-15]. The second approach defines regularity in function of the donation dates and their frequency; it classifies blood donors into (i) regular (≥2 donations within the last 24 months, the last one in the past 12 months), (ii) occasional (≥1 donations within the last 24 months, with none in the past 12 months) and (iii) inactive (no donation in the past 24 months) [12,16,17]. In omitting both the interval between the last two donations as well as the donors’ previous regularity, such classification overestimates the number of regular donors and restricts reactivation strategies, which can differ according to the anterior donor’s dynamic. Factors that encourage or interfere with voluntary blood donation, as well as donors’ retention, are multiple and complex. They may be personal (such as age, gender, education level, values, lifestyle, knowledge of the needs and of the system, social representations or medical contraindication), organisational (e.g. existence of mobile blood collections and organisation of the donors’ flow) or environmental (such as presence of donors in the entourage or site accessibility) [9,17-22]. Criteria of donors’ selection must be considered also, as they impact recruitment and retention. In the DR Congo, the main selection criteria are: age between 18 and 65 years old, a haemoglobin concentration >12g/dl, a body weight ≥50kg and an interval of at least 3 months between two donations [8].

African studies targeted so far the recruitment of donors and explored the sociodemographic profile, the epidemiological data, the information level as well as favourable and adverse attitudes toward voluntary blood donation [15]. They poorly explored the retention of voluntary donors and retention-associated factors. The context is similar in Bukavu, DR Congo, where the South-Kivu Provincial Blood Transfusion Centre (PBTC), namely our data collection site, is located [23]. The PBTC and its affiliated centres only cover a small part of transfusion needs, through mobile or fixed collections; the rest of the blood supply comes from replacement donations in hospitals [8,24]. As a result, a double public health concern arises, in essence (a) an increased morbidity and mortality of main transfusion recipients who are children less than 5 years old (75%) and women (15%) and (b) an increased residual risk of TTIs. Indeed, in the first half of 2018, 56% of maternal deaths in the DR Congo were caused by obstetrical haemorrhages [25]. The donors’ profile and retention (regularity) following the dynamics of voluntary donations, including affecting factors, are unknown to date in our study environment. Despite the shortage, this makes impossible any action targeting awareness of donation and re-awareness of former voluntary donors, as well as projected supply from voluntary donations, which are crucial steps for improving the blood supply. In order to fill that void, we performed a study in a single transfusion centre, the PBTC, located in South Kivu. We had two objectives: to describe the personal characteristics of donors who gave blood at least once in the PBTC and their donation behaviour between January 1^st^, 2015 and December 31^st^, 2017; and to determine the donation dynamics as well as the donors’ regularity rate in the same centre.

## Methods

**Study design and context:** this descriptive and analytical cross-sectional study included the records of blood donations performed in the South Kivu PBTC between January 1^st^, 2015 and December 31^st^, 2017. The PBTC is the technical body of the provincial coordination of the national blood transfusion programme. Its mission is to supply urban hospitals with blood. The first author is an extern collaborator and the third author is the doctor responsible for blood collections in the centre.

**Data sources:** a database compiling blood donors’ individual data and attributes of each donation was built from registry data and PBTC donation records. For each donor, notebooks gather the following information: name, age, gender, address, membership or not of a donors’ association (and if so, which one), date and place of donation, markers tested on the sample and test results. Six inventories covering the 3 year-study period were consulted, i.e. two registers per year: one for mobile collections and the other for collections on fixed site. For each donation, the record include the following information: donor’s characteristics (last name, surname, gender, address, occupation, education level and membership of an association), the donor’s history of donations, the medical, surgical and gynaeco-obstetrical history, the results of physical examination (body weight, blood pressure, pulse, breathing e.t.c.), decision on donation eligibility and when appropriate, the justification of temporary or permanent exclusion, post-donation reactions and their management. We accessed a total of 1,254 records, which corresponds to 773 voluntary donors over 3 years.

**Sample size:** the Schwartz formula below allowed calculating the minimal sample size, i.e. 345 donors: n=t^2^* pq/m^2^, n=sample size; t=95% confidence interval (CI) (Z=1.96); p=local prevalence of voluntary blood donation (estimated at 0.34 in DR Congo [4]); q=1-p=proportion of non-voluntary blood donors (=0.66); m=estimator’s degree of accuracy, fixed at 5% (0.05). Half of donation records, namely 627, were included in the study by drawing. After removing duplicates (N=10) and incomplete records (N=46), our final sample included 571 donation records, corresponding to 387 donors.

**Data mining:** the independent variables collected from registers and records included: age, body weight, gender, marital status, education level, occupation, degree of retention in 2017 (previous donor and newcomers), last donation date, membership or not of a donors’ association and collection site. Other variables were created from record data and registers, e.g. average distance between home and donation site, activity level in 2017 (active vs inactive) and interval between the last two donations. The last two variables are useful to assess regularity. The dependent variable, in other words the regularity profile, reflects the donors’ retention; it was created from data collected through a composite classification method, which combines both approaches described in the introduction. Regularity of previous donors and inter-donation interval were included as well. Such method is justified as existing approaches have limits we wanted to overcome, namely overestimating the number of regular donors ('retention') [11,12] and ignoring the regularity profile of previous donors who became inactive [11,15,16]. Our dependent variable was thus assessed through the following criteria (to which we added the anterior regularity): number of donations: ≥2 donations in the last 24 months (2016 and 2017); maximum interval of 12 months between the last two donations; date of the last donation <12 months (2017). These three variables were analysed to assess the previous regularity (that is, before 2017) and the regularity by the time of the study (2017-regularity). The following typology allowed the distinction of four types of donors: regular voluntary donors (group A): ≥2 donations during the last 24 months, with the last donation recorded in 2017 and an inter-donation interval <12 months; new voluntary donors (group B): first and single donation in 2017; previously regular voluntary donors (group C): 2 donations before 2017, less than 12 months apart; no donation in 2017. previously occasional voluntary donors (group D): ≥2 donations before 2017, with an inter-donation interval >12 months and no donation in 2017.

**Statistical analyses:** descriptive and comparative statistical analyses were performed with STATA software (version 15). Descriptive statistics of categorical data were presented in terms of relative frequencies while for continuous data, medians with inter-quartile ranges and proportions after categorisation were shown. Regular donors (group A) were compared with each group of non-regular donors (group B,C or D) and to all non-regular donors (groups B,C and D). Univariate analyses applied a student t test after logarithmic transformation and a (Pearson and Mc Nemar) Chi-2 test when applicable. In order to refine the search for characteristics associated with regularity, a multivariable logistic regression was performed; odds-ratios (ORs) and Wald test p-value will be presented. Any difference was considered as statistically significant for p<0.05 whereas confidence intervals are given at 95% (95%CI).

**Ethical statement:** the present research was approved by the South Kivu Provincial Direction of the National Health Ethics Committee (reference: CNES 001/DPSK/113PP/2018) and by the Ethics Committee of the UCLouvain/ Cliniques Universitaires Saint-Luc (reference: 2019/15JAN/001). Data collected from registers and donation records were anonymised.

## Results

The results section will be divided into three parts: donors’ characteristics and donation behaviour, donors’ regularity and factors associated with regularity.

**General characteristics of the donors:** sociodemographic characteristics, as well as those related to voluntary donations during the period of study are presented in Table 1. Among the 387 voluntary donors of the Centre, 82.2% were men (N=318), 60.5% were aged ≤25 years old (N=234), 73.1% were married (N=283), with no income, even if 54% of them (N=212) had reached the level of secondary education. Half the donors weighted ≤62kg and 3.3% of them (N=13) were below the minimum required weight (namely 50kg). More than 75% of former donors (84.8%; N=328) had already given blood at least once before 2017 and were members of a voluntary donors’ association. The large majority of donors were living in urban areas (85%; N=329); donors were going either to mobile or fixed collections in almost equal proportions. Three-quarters of the donors had to travel a maximum of 2km to give blood (Table 1).

**Table 1:** general characteristics of donors and voluntary donations in the South Kivu Provincial Blood Transfusion Centre

Variables [Min, Max]	Blood donors (N)	Proportion (%)	Median (P25-P75)
**Gender**			
Men	318	82.2	
Women	69	17.8	
**Age of donors [17,66]**			**24(21-31)**
≤ 25 years old	234	60.5	
26-45 years old	120	31.0	
≥ 46 years old	33	8.5	
**Education level**			
No school or Primary	49	12,7	
Secondary (high school)	212	54.8	
Higher education or university	126	32.5	
**Occupation**			
No income/no occupation	292	75.4	
Non-formal sector	53	13.7	
Formal sector (waged)	42	10.9	
**Body weight [42,135]**			**62(57-69)**
≤60kg	123	31.8	
61-69kg	173	44.7	
≥70kg	91	23.5	
**Member of an association**			
Members	338	87.3	
Independents	49	12.7	
**Living environment**			
Urban	329	85.0	
Rural	58	15.0	
**Degree of retention in 2017**			
Former donors	328	84.8	
New donors	59	15.2	
**Collection site**			
Mobile	191	49.4	
PBTC	188	48.6	
Combination of site	8	2.0	
Mean distance travelled (km) [0,20]	387	100.0	2(0-2)

Results concerning continuous variables are presented under the form of median and inter-quantile range (P25-P75); total number of blood donors=387; PBTC=Provincial Blood Transfusion Centre

**Regularity of the voluntary donors:** the donors’ activity was low in 2017: only 39% of donors gave blood that same year, although the mean inter-donation interval was ≤5 months for 50% of donors. Over the 3 year-period, the median number of donations per donor was 2; one donor gave 13 times. According to the classication method described above, 23.8% (N=92) of individuals were regular donors (group A) in 2017, while 41.6% (N=161) belonged to group C, i.e. donors regular before 2017 but who did not give blood in 2017. When comparing group C to group A (regular donors in 2017), there was a 72.8%-loss (N=161) of donors who were regular before 2017 (Mc Nemar Chi-2 test: 49.72; p<0.001). Only 27.2% (N=60 out of 221) of previously regular donors gave blood in 2017 and were considered as regular donors that same year. Among former regular donors, only 6.2% were discarded by the centre for medical reasons (N=10 out of 161); others had stopped donating blood for unknown reasons (Table 2).

**Table 2:** donation dynamics and donor regularity

Variables	N	Proportion (%)	Range (Min-Max)	Median (P25-P75)
**Donation dynamics**				
**Activity in 2017**				
Activity recorded	151	39		
No activity recorded	236	61		
Mean inter-donation interval (months)	235	61	1-52	5(3-8)
Missing*	152	39		
Number of donations per donor	387	100	1-13	2(1-2)
**Regularity profile in 2017**				
Regular donors	92	23.8		
New donors	59	15.2		
Former regular donors	161	41.6		
Former occasional donors	75	19.4		

Results concerning continuous variables are presented by their median and inter-quantile range (P25-P75); * donors who made only one donation during the study period

**Factors associated with donors’ regularity:** in this section, we mainly present the results of the comparison between 2017-regular donors (group A; N=92, i.e. 23.8%) and all non-regular donors (groups B, C and D; N=295, i.e. 76.2%) and previously regular donors (group C; N=161, i.e. 41.6%).

**Factors associated with regularity-univariate analysis (student t test and Pearson Chi-2):** regular donors (N=92, i.e. 23.8%) were in majority older (p<0.001), had a higher body weight (p<0.001), a mean higher number of donations (p<0.001), even though their inter-donation interval was longer (p<0.01), in comparison with all non-regular donors (N=295, i.e. 76.2%). As to previously regular donors (group C; N=161), they were younger and weighted less than regular donors; their inter-donation interval was also shorter and their average number of donations lower, compared to regular donors. Regardless of the regularity status, the mean distance travelled to the blood donation site was not statistically different when comparing all non-regular donors to previously regular donors (Table 3). Regular donors were more often married (p<0.01) and working in the formal sector (p<0.001) compared to non-regular donors. However, compared to former regular donors, a higher proportion of regular donors were women (p<0.01), had a higher or university education level (p<0.05) and worked in the formal sector (p<0.01) (Table 3).

**Table 3:** factors associated with regularity - univariate analysis (student t test and Pearson Chi-2)

a. Regularity of donors for continuous variables (student t test)					
**Variables**			**Variables**		
**Regular versus non-regular donors**	**Mean (95%CI)**	**P-value**	**Regular versus former regular donors**	**Mean (95%CI)**	**P-value**
**Age (years)**		**<0.0001**	**Age (years)**		**<0.01**
Regular (N=92)	30.7(28.3-33.2)		Regular (N=92)	30.7(28.3-33.2)	
Non-regular (N=295)	25.1(24.3-26)		Former regular (N=161)	26.7(25.5-28)	
**Body weight (kg)**		**<0.001**	**Body weight (kg)**		**<0.05**
Regular (N=92)	66.6(63.9-69.5)		Regular (N=92)	66.6(63.9-69.5)	
Non-regular (N=295)	62.1(61.1-63.2)		Former regular (N=161)	63.4(62-64.8)	
**Mean distance (km)**		**0.3422**	**Mean distance (km)**		**0.4203**
Regular (N=92)	2.8(2.5-3.2)		Regular (N=92)	2.8(2.5-3.2)	
Non-regular (N=295)	2.9(2.8-3.3)		Former regular (N=161)	2.9(2.6-3.2)	
**Mean interval (months)**		**<0.01**	**Mean interval (months)**		**<0.01**
Regular (N=92)	6.1(5.4-6.8)		Regular (N=92)	6.1(5.4-6.8)	
Non-regular (N=295)	4.9(4.5-5.4)		Former regular (N=161)	4.9(4.5-5.4)	
**Mean number of donations**		**<0.0001**	**Mean number of donations**		**<0.001**
Regular (N=92)	3.2(2.9-3.6)		Regular (N=92)	3.2(2.9-3.63)	
Non-regular (N=295)	1.4(1.3-1.5)		Former regular (N=161)	1.8(1.7-2)	
**b. Regularity of donors according to their characteristics-Pearson Chi-2 test (categorical variables)**					
**Variables**			**Variables**		
**Regular versus non-regular donors**	**Regular (%)**	**P-value**	**Regular versus former regular donors**	**Regular (%)**	**P-value**
**Gender**		**0.152**	**Gender**		**<0.01**
Men (N = 318)	22.3		Men (N = 216)	32.9	
Women (N = 69)	30.4		Women (N = 37)	56.8	
**Marital status**		**<0.01**	**Marital status**		**0.12**
Not married (N=318)	20.1		Not married (N=172)	33.1	
Married (N=104)	33.7		Married (N=81)	43.2	
**Association**		**0.554**	**Association**		**0.814**
Members (N=338)	24.3		Members (N=227)	36.1	
Independents (N=49)	20.4		Independents (N=26)	38.5	
**Education level**		**0.093**	**Education level**		**<0.05**
No education or primary (N=49)	14.3		No education or primary (N=38)	18.4	
Secondary (N=212)	22.6		Secondary (N=127)	37.8	
Higher or University (N=126)	29.4		Higher or University (N=88)	42.0	
**Occupation**		**<0.01**	**Occupation**		**<0.01**
Formal sector (N=42)	47.6		Formal sector (N=33)	60.6	
Non-formal sector (N=53)	18.9		Non-formal sector (N=43)	23.3	
No income/occupation (N=292)	21.2		No income/occupation (N=177)	35.0	
**Adress**		**0.205**	**Address**		**0.162**
Urban (N=329)	24.9		Urban (N=215)	38.1	
Rural (N=58)	17.2		Rural (N=38)	26.3	

**Factors associated with regularity-logistic regression:** the stepwise logistic regression highlighted the influence of three variables on regularity: donor age, gender and occupation. It confirmed the results of the univariate analysis for what age is concerned: indeed, the proportion of regular donors was higher among people aged >46 years old (OR=3.95; p<0.05). Besides, it highlighted a higher proportion of regular donors among women (OR=2.4; p<0.05). The professional status had an impact on donation regularity: workers of the formal sector accounted more regular donors compared to other professional categories (p<0.05). Even if not significantly different, a higher proportion of regular donors was observed among those whose inter-donation interval was between 3 and 12 months (OR=8.14, p=1368), compared to donors whose interval was shorter than 3 months (ORa=1) or above 12 months (Ora=7.27) (Table 4). When comparing regular donors and previously regular donors, results are similar: most regular donors were older, women, working in the formal sector.

**Table 4:** logistic regression of the 2017-frequency status, adjusted for donor age, gender and occupation

Variables	Regular			P value
	Proportion	Adjusted OR	95%CI	
**Gender**				**<0.05**
Men (N=318)	22.3	1		
Women (N=69)	30.4	2.4	1.11-5.17	
**Age**				**<0.05**
≤25 years old (N=234)	17.5	1		
26-46 years old (N=120)	25.8	1.04	0.55-2.13	
>46 years old (N=33)	60.6	3.95	1.5-10.4	
**Occupation**				**<0.05**
Formal sector (N=42)	47.6	1		
Non-formal sector (N=53)	18.9	0.2	0.07-0.61	
No income/occupation (N=292)	21.2	0.41	0.17-0.97	

Not included because non-significant: donors' body weight, marital status, education level and inter-donation interval

## Discussion

The present assessment of the voluntary donors’ characteristics at the centre highlighted an important loss of regular donors, while informing on some characteristics associated with regularity of donors from South Kivu (DR Congo). Our study reveals that regular voluntary donors are mostly mature women working in the formal sector. Characteristics of regular donors are overall in contrast with those of the whole population of donors over the study period, in essence mostly young men with no income.

**Low retention:** the rate of regular donors from 2017 (23.8%) was twice as low as in a previous study that focused on the residual risk in the same center, between 2010 and 2012 [14]. It was also lower than the rates observed in two multicentric studies carried out in French-speaking Africa in 2012 and 2017 [15,26]. In all these studies, donor regularity relied on the number of donations, without considering the last donation date during the last 12 months of the study, as recommended by other authors [12,16,17]. Thus, they probably overestimated the rate of regular donors. The low retention of voluntary donors was associated with a considerable loss of previously regular donors (72.8%); both are major risk factors for the transfusion safety in our province, which goes along a pre-existing lack of voluntary donations. As a result, a loss of quality and accessibility to transfusion care can jeopardize the health and even the life of main beneficiaries. The re-solicitation of previous donors who did not give blood in 2017 (former regular and former occasional, 61%; N=236/387) would have the advantage to limit the continuous recruitment efforts. Furthermore, their ITT rate would be lower compared to new donors [10,27], who must also be retained. Indeed, former donors were screened in the past and educated over at risk-behaviours to be avoided by blood donors.

**Impact of our composite classification method:** our composite classification method, that combines 4 parameters (number, frequency, inter-donation interval and previous regularity) allowed a better understanding of regularity and real donation dynamics compared to usual approaches; the latter have shown their limits in the assessment of donation dynamics [14,15] or skipped the donors’ previous regularity. Thus, the two usual classification methods might overestimate the forecasting of donations: the first one by relying on the number of donations only and the second one by not considering the regular donors’ inter-donation interval, which can vary and negatively impact the rate of supply. Consequently, the real availability is low [12,14,15]. By classifying donors into regular, occasional and inactive, without considering their past regularity [11,12,16,17], several profiles, useful for a re-awareness, might go unnoticed in the second classification. The reasons for stopping or spacing donations in time might differ, when comparing a former regular donor who became inactive or occasional to another donor who has never been regular; indeed, re-activation strategies will be different. The composite method we propose allows discarding donors who were categorised as regular by existing classifications, while creating opportunities for renewed awareness-raising in the future.

**Factors associated with the regularity of voluntary donors:** in our study, factors associated with donors’ regularity in 2017 corresponded in majority to factors mentioned in precious studies, namely advanced age [9,11,28,29], female gender [30] and waged employment [11,28,31]. The hypothesis would be that a long donation history of older volunteers allowed them to internalise the values around beneficiary donation and integrate blood donation in their lifestyle [22]. Mature women would be less susceptible to be pregnant or breastfeeding, which are two reasons for being temporarily excluded from blood donation. Nevertheless, even if the present study highlighted some characteristics associated with the profile of a regular donor, other complex factors, especially psychosocial (such as beliefs, motivation and values) that could explain the retention (or not) of blood donors still need to be resolved. The strengths of our study are the demonstration that donors’ regularity must be studied as a fully dependent variable in the local investigations performed in sub-Saharan transfusion services. Indeed, it is necessary to follow the donation dynamics, predict as much as possible the future donation flows and lay the foundation for a possible reactivation of former donors. By connecting, for the first time in sub-Saharan and Congolese regions, factors associated with current regularity of voluntary donation, we think the present study is original through its proposition of a classification method identifying groups to target in future recruitment campaigns, as well as groups of donors requiring more attention for their retention, i.e. younger donors and workers of the non-formal sector. Besides, our classification method allows considering the anterior regularity of donors who became non-regular, in order to investigate the reasons for loss of participation. This will help addressing the appropriate reactivation strategies. The present study only relied on the analysis of data collected from the centre registers and donation records; it’s main limit is the non-exploration of religious influence, representations around blood and blood donation and motivations for continuing or stopping blood donation. A qualitatitve study, through interviews and observation, will allow a better understanding of additional factors contributing to the stopping of blood donation or to the early return of some voluntary donors in order to make (re)-awereness campaigns more efficient.

## Conclusion

Our study highlights that combining different existing typologies to assess the regularity of voluntary blood donors provides a better understanding of donor retention by adding the donors' previous regularity and the interval between donations. This generates more accurate information and opening new avenues for awareness-raising, recruitment and retention of voluntary donors and a possible reactivation of previous donors to ensure enhanced safety of transfusions.

### What is known about this topic

- It is well known that the supply of blood donations depends on the recruitment and retention of donors;
- To date, various methods to assess blood donor retention and characteristics are associated with that retention in developed countries;
- Donor regularity assessment allows tracking of risk profiles for donor abandonment and reactivation of inactive donors.

### What this study adds

- This study quantifies for the first time the regularity of voluntary blood donors in sub-Saharan Africa;
- The proposed composite approach allows a better understanding of donor retention, particularly in sub-Saharan Africa;
- This study opens up new avenues for awareness-raising, recruitment and possible reactivation of previous donors.
